# Progress and Prospects of the Buffalo Medical College

**Published:** 1879-04

**Authors:** James P. White


BUFFALO

### Medical and Surgical Journal.

VOL. XVIII. APRIL, 1879. NO. 9.

### Original Communications.

:o:

ART. I.—*Progress and Prospects of the Buffalo Medical College.* A speech at the Banquet of the Alumni Association in response to the toast of Alma Mater. By Prof. JAMES P. WHITE, M. D.

*Mr. President and Gentlemen of the Alumni Association:*

For more than a generation I have used my best energies in the interest of the Buffalo Medical College, and have never refused any call in her name; and it is certainly too late for me to fail her now, especially as her hold upon my affections grows stronger and stronger with each succeeding year.

I am not expected to make a set speech in answer to the compliment you have just extended to this object of our affections; therefore I shall have but little to say on this interesting occasion. As the oldest member of the present faculty, and the only one of the founders now connected with the institution, I cannot refrain from a few words of history of your Alma Mater and of her prospects, and the desires and intentions of the faculty for her preservation and improvement. A little more than a third of a century ago—in the winter of 1845–6—Dr. Austin Flint, with the speaker, aided by a few such public spirited citizens as Millard Fillmore, N. K. Hall, O. H. Marshall and George R. Babcock, procured a charter for a University in the city of Buffalo, then just emerging from the obscurity of a "western town." We, Dr. F. and myself, were soon joined by Prof. Frank H. Hamilton, of the Geneva Medical School, who subsequently induced his colleagues of that institution to become interested in our project. We, therefore, commenced teaching medicine with able and for the most part experienced lecturers. We assembled a class of sixty or more students in the old Baptist Church, modified to suit our purposes, then standing on the corner of Washington and Seneca streets, on the site of the present Postoffice building; and at commencement, conferred the degree of Doctor in Medicine upon seventeen young men, of whom, those now living are almost "old men." Thus was established the first successful educational institution, above the grade of common school, in western New York, after the Senecas left to us their homes and lands. During the year 1849, unaided by state bounty, which had been extended to all its other colleges, chiefly through the generosity of the citizens of this then small and struggling city, and through the energetic efforts of two or three of the Faculty, the present substantial and convenient edifice was erected and dedicated to the cause of medical education. In parentheses permit me to say, that it behooves the friends of education in this now large and flourishing city, to make strenuous efforts that the remaining departments provided for in the charter, and especially the academic and law, be organized without further delay. We have now more than six times the population, and twenty times the wealth (assessed value), we had when the charter was obtained and the medical department carried into successful operation, and it should be an easy matter to place the whole University in a flourishing condition, capable of teaching all our own young men, as well as those of the neighboring country, to take a wise and intelligent care of all these great interests which soon, in the course of nature, must be committed to their charge. It is fully time that the men of wealth and enterprise should interest themselves more actively in higher education, and provide our city with colleges as well as parks, public school and other municipal buildings. It is conceded that our future as a nation, and the success of our democratic institutions, depends upon the higher education of the youth of the Republic, and the intelligent interest they shall take in public affairs. We must all do our part, and I am confident that it requires only energy and a proper presentation of the subject to our men of wealth and enterprise, to accomplish this great desideratum for our beautiful city. From the day of graduation of this little band of doctors our success as a school was assured; and if the members of the Faculty were true to themselves it became a question of degree only. (No pun intended.) Although the project was met with bitter opposition and we were surrounded by cheaper schools, our progress has been steadily "onward and upward," and the session just closed has been the most successful in our history. The Buffalo Medical College now has its graduates scattered throughout the entire country and indeed over the whole civilized world, and I am happy to state that they stand well in the profession of their choice, and are men of progress and position in the community, doing honor to Alma Mater. It is our boast, and I take pleasure in here repeating it, that of the numerous applicants for positions in the army and navy during the late war, not a graduate of this college was rejected by any examining board. In my journeying from the Atlantic to the Pacific and the gulf of St. Lawrence to the gulf of Mexico, I have never met one of our representatives that I did not receive a hearty welcome, and that my heart did not go out to him as to no other man. There exists between us a Free Mason like understanding and cordiality, an assured confidence which can be engendered only by the relations which we have held toward each other of teacher and pupil.

Meantime my early associates have fallen by the way or have been transferred to other fields of usefulness, and I am left alone of the originators and first faculty. The vacancies, as they have occurred, have been filled by able and progressive men, and I do not hesitate to state that didactic medicine is as well taught to-day in the University of Buffalo as in any institution in the land. In many things essential to the thorough teaching of medicine this school has taken the lead; here clinical midwifery was first introduced into America, before the graduating class of 1850, and the neophite learned his duties at the bedside of the parturient, where alone it is possible to acquire that knowledge. The speaker, in conjunction with the late Bishop Timon, established a lying-in hospital for the permanent use of our pupils, but circumstances have taken it out of our reach for the present. Physiology was first taught experimentally at this school by Dalton more than a quarter of a century since, who with his pupil and successor here, Flint, Jr., and his pupil and successor Mason, are to-day the advanced men of that department in America, and stand at its head. In an effort to extend the length of the lecture course, made many years since, we were among the few who adopted that improvement. We early advocated in the American Medical Association the rejection of certificates of study from irregular practitioners. During discussions in the American Medical Association, on the subject of medical education, it has often been asserted that nothing would so much contribute to the elevation of the standard, as the establishment of some system for examination of candidates for degrees by competent persons not connected with the faculty. This has always been the custom at this College. Believing, at its organization, that the interests of the profession and the public would be promoted by an independent Board of Examiners, who, without fee and without sympathy to bias their judgment, should stand between the Faculty and the students as guardians of the rights and interests of both, we instituted the Board of Curators, selected from the most able and distinguished members of the profession throughout all the neighboring country as well as this city. So far as I am aware no other school has this impartial tribunal, and I think that too great importance can scarce be attached to the utility and merit of the system where, as is the case here, the members are selected from a large area of country and are the most prominent and progressive men, unbiased in judgment and influenced only by a desire for the best interests of all. If, for any reason, the Faculty should recommend unsuitable persons, this Board of Revision would be very likely to detect their failings and throw them out. Besides which, this arrangement serves to make the Faculty more cautious and thorough in their own examinations, knowing, as they do, that their work will be subjected to sharp criticism and any flaws or weakness detected. Perhaps we may in some measure attribute to this fact the superiority of our classes in the aggregate. These are a few of our "good works" past; now for the future.

We propose to extend the period of instruction from sixteen to twenty weeks, beginning with the next course. In order to bring teacher and pupil into more intimate relations, we assume most cheerfully, the additional labor of giving several evenings in each week to extra instruction in the form of "quiz" or review of the subjects taught in the regular lectures. These reunions or reviews will be informal, free of extra charge, and will give the pupil an opportunity of asking explanation of any points that may not be clear to his own mind; and going over the subjects already placed before him will serve to impress them more firmly on his memory. Who can so well explain the various topics discussed in the lectures, as the professor who has given the subject much thought and special study in his own department? He, also, can best clear up obscure passages in the text books. Not the least recommendation of this system of informal "evenings at home," as they might be called, is the fact that teacher and scholar will be brought to know each other more intimately as they meet without the restraint necessarily observed in the more formal lecture. To those who desire to learn to make practical analyses in chemistry and the use of the microscope, further facilities will be afforded. Professors Doremus and Mason will examine and quiz the class evenings the same as the other professors, according to the above plan, and in addition will receive pupils in the laboratory for special instruction in practical, experimental chemical work, and demonstrations in experimental physiology and microscopic anatomy, which are impossible to exhibit in the lecture room.

Their lectures and experiments before the class will be as full and complete as it is possible to make them, but for this additional work, with the able professors in charge of those two departments, special classes will be made for which they will charge a small fee. These classes in this instruction are entirely voluntary on the part of the pupil, and the facilities are offered only to those who desire more minute teaching than it is possible to obtain at a class lecture.

In my inaugural address as President of the New York State Medical Society in 1870, will be found the following remarks on medical instruction:

"The medical schools of the State, may now be said to be in a highly satisfactory condition. They are yearly increasing their requirements for graduation—though the standard is undoubtedly still too low—demanding constant effort on the part of all men of progress, to carry it still higher.

"The different institutions are yearly enlarging their facilities for instruction, and in laudable competition for increased classes, are affording opportunities, in both didactic and clinical teaching, which the student will scarcely find surpassed in any part of Europe.

"There is, however, one step in a forward direction which should now be taken, and which it seems to me this society can promote by its influence and endorsement.

"I allude to the teaching of Psychological Medicine, as a part of the curriculum of the college course. Is it less important that the diseases of the mind, always involving the brain and nervous system, with their sympathetic connexions and influences, should be properly taught and illustrated with cases, than any of those maladies now deemed essential to be clinically taught, in every institution? The attention of the profession is beginning to receive this direction—some steps have already been taken toward its accomplishment, and I believe the medical mind is thoroughly ripe for action, if commended by this society in suitable and proper terms. Permit me therefore to suggest the appointment of a select committee, to take this matter duly into consideration, and recommend to the society, what action, if any, it should take in its furtherance."

The society, upon the report of a committee, of which that able alienist, Dr. John P. Gray, was chairman, after careful consideration, unanimously recommended to the schools of the state that they give instruction upon this important subject. Following up this initiatory movement in the state society, I subsequently brought the matter before the American Medical Society, where it received a hearty endorsement. Some of the Metropolitan schools have already acted upon this suggestion, and appointed professors to teach Psychological Medicine as a special department. It is with great satisfaction that I assure you this long cherished desire on my part is likely soon to be an accomplished fact in your Alma Mater. We shall, I trust, at an early day, have an institution opened here that will furnish ample opportunity for clinical instruction, and there is little doubt that the enterprising faculty of this college will make it available in furthering progressive education. Insanity is a subject of which the young practitioner is generally totally ignorant; but I trust, that at no distant day, the graduates of this college will leave it thoroughly instructed therein. It is needless to state that the clinical advantages afforded by the two large hospitals, situate as they are in a city of nearly 200,000 inhabitants, afford ample opportunity for seeing all the diseases found in this country, and without such a crowd as to prevent the modest from examining patients and witnessing operations. We, the faculty, claim for the Buffalo Medical College that it has always been progressive—intelligently, conservatively progressive—in everything pertaining to medical instruction and that it will continue in the same course. We recognize the fact that modern medicine consists more in preventive than curative measures; we shall enlarge our course in hygiene and sanitary measures and abridge it in dry therapeutical detail; we shall offer to our pupils an opportunity to profit by all the advances in diagnosis whether resulting from new instrumental devices, new applications of recognized principles, or in new methods of chemical analysis of the various secretions of the human body, or of the elements taken for nutrition, alimentation or medication. In short, we shall use every means in our power to send out our graduates thoroughly armed and equipped for the contest with disease, and you may rest assured that your Alma Mater will always deserve your confidence and support. It shall be our aim to raise higher and still higher the standard of medical education, and secure the confidence and affection of all those who have shown themselves worthy of the honors of the Buffalo Medical College.

The remarks of Prof. White were loudly applauded.

